# DNA methylation-based measures of biological age: meta-analysis predicting time to death

**DOI:** 10.18632/aging.101020

**Published:** 2016-09-28

**Authors:** Brian H. Chen, Riccardo E. Marioni, Elena Colicino, Marjolein J. Peters, Cavin K. Ward-Caviness, Pei-Chien Tsai, Nicholas S. Roetker, Allan C. Just, Ellen W. Demerath, Weihua Guan, Jan Bressler, Myriam Fornage, Stephanie Studenski, Amy R. Vandiver, Ann Zenobia Moore, Toshiko Tanaka, Douglas P. Kiel, Liming Liang, Pantel Vokonas, Joel Schwartz, Kathryn L. Lunetta, Joanne M. Murabito, Stefania Bandinelli, Dena G. Hernandez, David Melzer, Michael Nalls, Luke C. Pilling, Timothy R. Price, Andrew B. Singleton, Christian Gieger, Rolf Holle, Anja Kretschmer, Florian Kronenberg, Sonja Kunze, Jakob Linseisen, Christine Meisinger, Wolfgang Rathmann, Melanie Waldenberger, Peter M. Visscher, Sonia Shah, Naomi R. Wray, Allan F. McRae, Oscar H. Franco, Albert Hofman, André G. Uitterlinden, Devin Absher, Themistocles Assimes, Morgan E. Levine, Ake T. Lu, Philip S. Tsao, Lifang Hou, JoAnn E. Manson, Cara L. Carty, Andrea Z. LaCroix, Alexander P. Reiner, Tim D. Spector, Andrew P. Feinberg, Daniel Levy, Andrea Baccarelli, Joyce van Meurs, Jordana T. Bell, Annette Peters, Ian J. Deary, James S. Pankow, Luigi Ferrucci, Steve Horvath

**Affiliations:** ^1^ Longitudinal Studies Section, Translational Gerontology Branch, Intramural Research Program, National Institute on Aging, National Institutes of Health, Baltimore, MD 21224, USA; ^2^ The NHLBI's Framingham Heart Study, Framingham, MA 01702, USA; ^3^ Population Sciences Branch, Division of Intramural Research, National Heart, Lung, and Blood Institute, National Institutes of Health, Bethesda, MD 01702, USA; ^4^ Centre for Cognitive Ageing and Cognitive Epidemiology, University of Edinburgh, 7 George Square, Edinburgh, EH8 9JZ, UK; ^5^ Medical Genetics Section, Centre for Genomic and Experimental Medicine, Institute of Genetics and Molecular Medicine, University of Edinburgh, Edinburgh, EH4 2XU, UK; ^6^ Queensland Brain Institute, University of Queensland, Brisbane, QLD, Australia; ^7^ Laboratory of Environmental Epigenetics, Departments of Environmental Health Sciences and Epidemiology, Columbia University Mailman School of Public Health, New York, NY 10032, USA; ^8^ Department of Internal Medicine, Erasmus University Medical Centre, Rotterdam, 3000 CA, The Netherlands; ^9^ Institute of Epidemiology II, Helmholtz Zentrum München, 85764 Neuherberg, Germany; ^10^ Department of Twin Research and Genetic Epidemiology, Kings College London, London SE1 7EH, UK; ^11^ Division of Epidemiology and Community Health, University of Minnesota, Minneapolis, MN 55455, USA; ^12^ Division of Biostatistics, University of Minnesota School of Public Health, Minneapolis, MN, 55455, USA; ^13^ Human Genetics Center, School of Public Health, University of Texas Health Sciences Center at Houston, Houston, TX, USA; ^14^ Human Genome Sequencing Center, Baylor College of Medicine, Houston, TX, USA; ^15^ Center for Epigenetics, Johns Hopkins University, Baltimore, MD 21205, USA; ^16^ Department of Medicine, Beth Israel Deaconess Medical Center and Harvard Medical School, Boston, MA, USA; ^17^ Institute for Aging Research, Hebrew Senior Life, Boston, MA 02215, USA; ^18^ Department of Epidemiology, Harvard School of Public Health, Boston, MA 02115, USA; ^19^ Department of Biostatistics, Harvard School of Public Health, Boston, MA 02115, USA; ^20^ Department of Biostatistics, Boston University School of Public Health, Boston, MA 02118, USA; ^21^ Section of General Internal Medicine, Department of Medicine, Boston University School of Medicine, Boston, MA 02118, USA; ^22^ Geriatric Unit, Usl Centro Toscana, Florence, Italy; ^23^ Laboratory of Neurogenetics, Intramural Research Program, National Institute on Aging, National Institutes of Health, Bethesda, MD 20814, USA; ^24^ Epidemiology and Public Health, Medical School, University of Exeter, RILD, Exeter EX2 5DW, UK; ^25^ Research Unit of Molecular Epidemiology, Helmholtz Zentrum München, 85764 Neuherberg, Germany; ^26^ Institute of Health Economics and Health Care Management, Helmholtz Zentrum München, 85764 Neuherberg, Germany; ^27^ Division of Genetic Epidemiology, Department of Medical Genetics, Molecular and Clinical Pharmacology, Innsbruck Medical University, Innsbruck 6020, Austria; ^28^ Institute for Biometrics and Epidemiology, German Diabetes Center, Leibniz Center for Diabetes Research at Heinrich Heine University, 40225 Düsseldorf, Germany; ^29^ University of Queensland Diamantina Institute, University of Queensland, Brisbane, Queensland, Australia; ^30^ Department of Epidemiology, Erasmus University Medical Centre, Rotterdam, 3015 CN, The Netherlands; ^31^ HudsonAlpha Institute for Biotechnology, Huntsville, AL 35806, USA; ^32^ Department of Medicine, Stanford University School of Medicine, Stanford, CA 94305, USA; ^33^ Human Genetics, David Geffen School of Medicine, University of California Los Angeles, Los Angeles, CA 90095, USA; ^34^ VA Palo Alto Health Care System, Palo Alto CA 94304, USA; ^35^ Department of Preventive Medicine, Feinberg School of Medicine, Northwestern University Chicago, IL 60611, USA; ^36^ Robert H. Lurie Comprehensive Cancer Center, Feinberg School of Medicine, Northwestern University Chicago, IL 60611, USA; ^37^ Department of Medicine, Brigham and Women's Hospital, Harvard Medical School, and the Department of Epidemiology, Harvard T.H. Chan School of Public Health, Boston, MA 02215, USA; ^38^ Center for Translational Science Children's National Medical Center, George Washington University Washington, DC 20010, USA; ^39^ Department of Family Medicine and Public Health, University of California-San Diego, La Jolla, CA 92093-0725, USA; ^40^ Department of Epidemiology, University of Washington School of Public Health, Seattle, WA 98195, USA; ^41^ Public Health Sciences Division, Fred Hutchinson Cancer Research Center, Seattle, WA 98109, USA; ^42^ Departments of Medicine, Molecular Biology/Genetics, Oncology, and Biostatistics, Johns Hopkins University School of Medicine, Baltimore, MD 21205, USA; ^43^ Population Sciences Branch, Division of Intramural Research, National Heart, Lung, and Blood Institute, National Institutes of Health, Bethesda, MD 01702, USA; ^44^ Department of Environmental Health, Harvard T.H. Chan School of Public Health, Boston, MA 02115, USA; ^45^ Department of Psychology, University of Edinburgh, 7 George Square, Edinburgh, EH8 9JZ, UK; ^46^ Department of Biostatistics, School of Public Health, University of California Los Angeles, Los Angeles, CA 90095, USA

**Keywords:** all-cause mortality, lifespan, epigenetics, epigenetic clock, DNA methylation, mortality

## Abstract

Estimates of biological age based on DNA methylation patterns, often referred to as “epigenetic age”, “DNAm age”, have been shown to be robust biomarkers of age in humans. We previously demonstrated that independent of chronological age, epigenetic age assessed in blood predicted all-cause mortality in four human cohorts. Here, we expanded our original observation to 13 different cohorts for a total sample size of 13,089 individuals, including three racial/ethnic groups. In addition, we examined whether incorporating information on blood cell composition into the epigenetic age metrics improves their predictive power for mortality. All considered measures of epigenetic age acceleration were predictive of mortality (p≤8.2×10^−9^), independent of chronological age, even after adjusting for additional risk factors (p<5.4×10^−4^), and within the racial/ethnic groups that we examined (non-Hispanic whites, Hispanics, African Americans). Epigenetic age estimates that incorporated information on blood cell composition led to the smallest p-values for time to death (p=7.5×10^−43^). Overall, this study a) strengthens the evidence that epigenetic age predicts all-cause mortality above and beyond chronological age and traditional risk factors, and b) demonstrates that epigenetic age estimates that incorporate information on blood cell counts lead to highly significant associations with all-cause mortality.

## INTRODUCTION

DNA methylation-based biomarkers, often referred to as “epigenetic age” or “epigenetic clock”, are robust estimators of chronological age of an individual [1–4]. For example, a measure of epigenetic age based on levels of methylation in 353 CpG dinucleotide markers (cytosine linked to guanine by a phosphate group) allow the estimation of the age of an individual. This estimate is consistent across most types of biological specimens, including whole blood, brain, breast, kidney, liver, lung, and saliva and cell types, including CD4+ T cells, monocytes, B cells, glial cells, and neurons [3].

Recent studies suggested that epigenetic age is associated with age-related health outcomes above and beyond chronological age. For example, we and others have shown that individuals whose epigenetic age was greater than their chronological age (i.e., individuals exhibiting epigenetic “age acceleration”) were at an increased risk for death from all causes, even after accounting for known risk factors [5–7]. Further, we recently showed that the offspring of semi-supercentenarians (subjects who reached an age of 105-109 years) have a lower epigenetic age than age-matched controls [8]. Based on these findings, it has been hypothesized that epigenetic age captures some aspect of biological age and the resulting susceptibility to disease and multiple health outcomes. A first step in testing this hypothesis is to test whether epigenetic age predicts longevity in multiple populations and across ethnic groups.

In many studies epigenetic age is estimated from DNA derived from blood samples. It is well known that blood cell composition changes with age and some of these changes might be independent predictors of mortality [9–12]. Thus, it is of interest to understand whether considering information on blood cell composition in measures of epigenetic age improves their predictive power for mortality.

Here, we evaluated the ability to predict time to death for blood-based epigenetic age measures, both published and novel measures that incorporate information on blood cell composition. Due to the well documented age-related changes in blood cell composition, we distinguished epigenetic measures of age that were independent of changes in blood cell composition (cell-intrinsic measures), and measures that incorporated age-related changes in blood cell composition (“extrinsic” measures). By increasing the number of independent cohort studies, we more than doubled the number of mortality events available for analysis, which allowed for detailed subgroup analyses including those based on race/ethnicity.

## RESULTS

### Cohort studies

Our meta-analysis included 13 population-based cohorts. An overview of the cohorts is provided in Table 1. Our study involved 3 racial/ethnic groups: non-Hispanic whites (n=9,215), Hispanics (n=431), and Blacks (n=3,443). Detailed descriptions of each cohort can be found in the Supplemental Materials.

**Table 1:** Baseline characteristics of participating cohorts

Cohort	N	N_deaths_ (%)	Follow-up duration (years)*	Age (years)*	r_Horvath_^†^	r_Hannum_ ^‡^
1. WHI (White)	995	309 (31%)	15.4 (14.0-16.4)	68 (65-72)	0.67 (p=5.1×10^−131^)	0.73 (p=8.0×10^−167^)
2. WHI (Black)	675	176 (26%)	15.4 (13.7-16.5)	62 (57-67)	0.70 (p=1.2×10^−100^)	0.76 (p=3.0×10^−128^)
3. WHI (Hispanic)	431	78 (18%)	15.2 (14.1-16.3)	61 (56-67)	0.78 (p=8.9×10^−90^)	0.79 (p=1.3×10^−93^)
4. LBC 1921	445	312 (70%)	10.2 (6.2-12.9)	79 (78-79)	0.15 (p=1.5×10^−3^)	0.13 (p=6.0×10^−3^)
5. LBC 1936	919	106 (12%)	7.5 (6.9-8.4)	69 (68-70)	0.15 (p=4.9×10^−6^)	0.16 (p=1.1×10^−6^)
6. NAS	647	221 (34%)	11.6 (8.6-12.9)	72 (68-77)	0.69 (p=1.3×10^−92^)	0.76 (p=8.2×10^−123^)
7. ARIC (Black)	2,768	1,075 (39%)	20.3 (14.3-21.4)	57 (52-62)	0.65 (p<1×10^−200^)	0.71 (p<1×10^−200^)
8. FHS	2,614	236 (9%)	6.2 (5.6-6.9)	66 (60-73)	0.84 (p<1×10^−200^)	0.86 (p<1×10^−200^)
9. KORA	1,257	42 (3%)	4.4 (4.0-4.8)	61 (54-68)	0.84 (p<1×10^−200^)	0.88 (p<1×10^−200^)
10. InCHIANTI	506	91 (18%)	15.0 (14.6-15.5)	67 (57-73)	0.82 (p=3.2×10^−124^)	0.85 (p=2.1×10^−142^)
11. Rotterdam	710	32 (5%)	5.6 (5.3-5.8)	58 (54-62)	0.72 (p=1.9×10^−114^)	0.76 (p=1.3×10^−134^)
12.Twins UK	805	30 (4%)	8.5 (7.5-8.5)	58 (51-65)	0.87 (p<1×10^−200^)	0.89 (p<1×10^−200^)
13. BLSA (white)	317	26 (8%)	5.3 (4.0-6.6)	66 (58-73)	0.85 (p=1.1×10^−89^)	0.88 (p=7.2×10^−104^)
**Total**	**13,089**	**2734 (21%)**				

The last 3 columns report robust correlation coefficients (biweight midcorrelation) between chronological age and two epigenetic age estimates (Horvath and Hannum).

* Median (25^th^ percentile - 75^th^ percentile)

† Biweight midcorrelation coefficient of chronological age with epigenetic age using the Horvath method.

‡ Biweight midcorrelation coefficient of chronological age with epigenetic age using the Hannum method.

### Epigenetic age estimation

We used two methods for estimating the epigenetic age of each blood sample (Table 2). First, we used the approach by Horvath (2013) based on 353 CpGs, as described in [3] and Methods. Second, we used the approach by Hannum et al. (2013) based on 71 CpGs [2]. Both epigenetic age estimates were correlated with chronological age at the time of blood draw (Table 1) with biweight midcorrelation coefficients ranging from 0.65 to 0.89. But birth cohorts were excluded from this correlation analysis because it is not meaningful to calculate correlations with chronological age in this situation. The Horvath and Hannum estimates were also highly correlated with each other (r=0.76) even though the underlying sets of CpGs share only 6 CpGs in common. (Supplementary Table 1).

**Table 2:** Overview of various measures of epigenetic age acceleration

Measure of age acceleration	Short name of measure	Epigenetic age estimate	Uses blood counts	Correlation with blood counts	Conserved in breast tissue
(Universal) epigenetic age acceleration	*AgeAccel_Horvath_ (AgeAccel)*	Horvath: 353 CpGs	no	weak	yes
Intrinsic epigenetic age acceleration (Horvath)	*IEAA.Horvath (IEAA)*	Horvath: 353 CpGs	yes	very weak	yes
Age acceleration based on Hannum	*AgeAccel_Hannum_*	Hannum: 71 CpGs	no	moderate	no
Intrinsic epigenetic age acceleration (Hannum)	*IEAA.Hannum*	Hannum: 71 CpGs	yes	very weak	no
Extrinsic epigenetic age acceleration	*EEAA*	Enhanced Hannum	yes	strong	no

**Figure 1:**
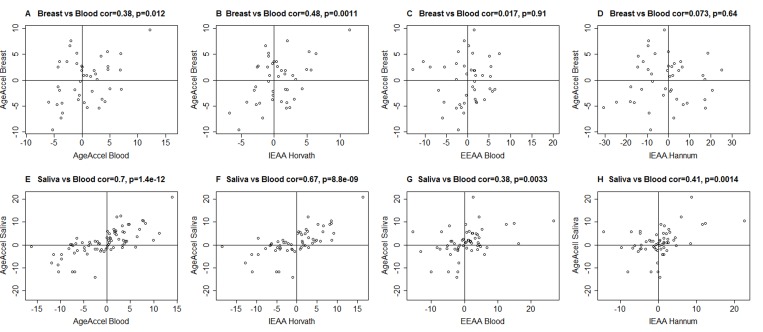
Epigenetic age acceleration in blood versus that in breast or saliva (**A**-**D**) Epigenetic age acceleration in healthy female breast tissue (y-axis) versus various measures of epigenetic age acceleration in blood: (**A**) universal measure of age acceleration in blood, (**B**) intrinsic epigenetic age acceleration based on the Horvath estimate of epigenetic age, (**C**) extrinsic epigenetic age acceleration, (**D**) intrinsic epigenetic age acceleration based on the Hannum estimate of epigenetic age. (**E**-**H**) analogous plots for epigenetic age acceleration in saliva (y-axis) and (**E**) *AgeAccel*, (**F**) *IEAA* based on Horvath, (**G**) *EEAA*, (**H**) *IEAA* based on the Hannum estimate. The y-axis of each plot represents the universal measure of age acceleration defined as the raw residual resulting from regressing epigenetic age (based on Horvath) on chronological age.

Description of the differences between epigenetic age and age acceleration measures. Column “Correlation with blood counts” relates to Supplementary Table 4. Column “Conserved in breast tissue” relates to Figure 1

### Estimated blood cell counts that relate to chronological age

We estimated the abundance of ten blood cell types based on observed DNA methylation patterns (Methods) –exhausted/senescent CD8+ T cells (CD8+CD28−CD45RA−), CD8+ naïve, CD8+ total, CD4+ naïve, CD4+ total, natural killer cells, B cells, monocytes, granulocytes, and plasmablasts. To study age-related changes in blood cell composition, we correlated these estimated blood cell counts with chronological age in all of the cohort studies (Supplementary Table 2). Our results are congruent with findings from flow cytometric studies that demonstrate that the abundance of naïve CD8+ T cells decreases with age (reflecting thymic involution), whereas exhausted/senescent CD8+ T cells increase with age [9–12].

### Measures of epigenetic age acceleration

Despite high correlations, epigenetic age can deviate substantially from chronological age at the individual level. The difference between epigenetic age and chro nological age can be used to define “delta age” but the resulting measure exhibits a negative correlation with chronological age. By contrast, all of our measures of epigenetic age acceleration are defined such that they are uncorrelated with chronological age.

An overview of several measures of epigenetic age acceleration is presented in Table 2. One such measure (denoted as *AgeAccel*) is defined as the residual that results from regressing epigenetic age on chronological age. Thus, a positive value of *AgeAccel* indicates that the epigenetic age is higher than expected, based on chronological age. These Horvath and Hannum based measures of age acceleration are denoted by *AgeAccel_Horvath_* and *AgeAccel_Hannum_*, respectively. For the sake of brevity and consistency with other publications from our group, we abbreviate *AgeAccel_Horvath_* as *AgeAccel*.

*AgeAccel_Hannum_* and to a lesser extent *AgeAccel* were previously shown to correlate with blood cell counts [5]. Thus, we distinguished two broad categories of measures of epigenetic age acceleration when dealing with DNA methylation from blood or peripheral blood mononuclear cells (PBMCs): intrinsic and extrinsic epigenetic measures, which are independent of, or enhanced by blood cell count information, respectively. We define *intrinsic* epigenetic age acceleration (*IEAA*) as the residual resulting from regressing epigenetic age on chronological age and measures of blood cell counts (Methods). By definition, *IEAA* is not correlated with chronological age and is weakly correlated with estimated measures of blood cell counts (Supplementary Table 4). *IEAA* is meant to capture cell-intrinsic properties of the aging process that exhibit some preservation across various cell types and organs. Compared to our other measures of age acceleration, *IEAA*, adapted from the Horvath measure of epigenetic age, exhibited significant correlations with epigenetic age acceleration in breast tissue (r=0.48, p=0.0011, Figure 1B) and saliva (r=0.67, p=8.8×10^−9^, Figure 1F). By contrast, an analogous measure of *IEAA* based on the Hannum measure showed much weaker correlations (r=0.073 in breast and r=0.41 in saliva Figure 1D, 1H). For this reason, we focused on the Horvath measure of *IEAA*.

The age-related changes to blood cell composition (Supplementary Table 4) can be leveraged to capture aspects of immunosenescence. Using these measures, we derived a novel extrinsic epigenetic age acceleration (*EEAA*) measure by up-weighting the blood cell count contributions of *AgeAccel_Hannum_* (Methods and Supplementary Table 4).

Descriptive statistics (minimum, maximum, median) of the measures of epigenetic age acceleration can be found in Supplementary Table 3.

### Cox regression models of all-cause mortality

We used Cox regression models to assess the predictive value of our measures of epigenetic age acceleration for all-cause mortality. All of our Cox models were adjusted for the age at baseline (blood draw). Additional multivariate models further adjusted for covariates assessed at baseline (chronological age, body mass index, educational level, alcohol intake, smoking pack-years, prior history of diabetes, prior history of cancer, hyper-tension status, self-reported recreational physical activity).

Our novel measure of extrinsic age acceleration *EEAA* led to smaller p-values for the associations with all-cause mortality than the original measure *AgeAccel_Hannum_* in univariate Cox models (p_*EEAA*_=7.5×10^−43^, p_*AgeAccelHannum*_=1.4×10^−34^, Supplementary Figure 1) and in multivariate Cox models (p_*EEAA*_=3.4×10^−19^, p_*AgeAccelHannum*_=6×10^−15^, Supplementary Figure 2). Further, when both *EEAA* and *AgeAccel_Hannum_* were included in the same Cox model, only *EEAA* remained significant in the WHI data and FHS univariate models. Since these results indicate that *EEAA* outperforms the closely related measure *AgeAccel_Hannum_* when it comes to mortality prediction, we removed the latter from subsequent analyses.

**Figure 2:**
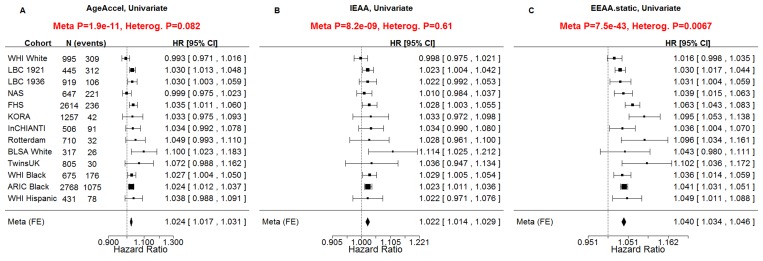
Univariate Cox regression meta-analysis of all-cause mortality A univariate Cox regression model was used to relate the censored survival time (time to all-cause mortality) to (**A**) the universal measure of age acceleration (*AgeAccel*), (**B**) intrinsic epigenetic age acceleration (*IEAA*), (**C**) extrinsic epigenetic age acceleration (*EEAA*). The rows correspond to the different cohorts. Each row depicts the hazard ratio and a 95% confidence interval. The coefficient estimates from the respective studies were meta-analyzed using a fixed-effect model weighted by inverse variance (implemented in the *metafor* R package [34]). It is not appropriate to compare the hazard ratios and confidence intervals of the different measures directly because the measures have different scales/distributions. However, it is appropriate to compare the meta analysis p values (red sub-title of each plot). The p-value of the heterogeneity test (Cochran's *Q*-test) is significant if the cohort-specific estimates differed substantially.

**Figure 3:**
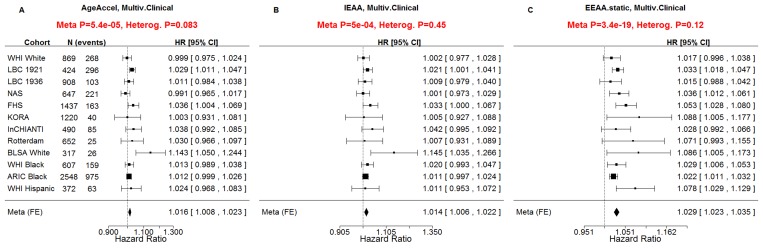
Multivariate Cox regression meta-analysis adjusted for clinical covariates A multivariate Cox regression model was used to relate the censored survival time (time to all-cause mortality) to (**A**) the universal measure of age acceleration (*AgeAccel*), (**B**) intrinsic epigenetic age acceleration (*IEAA*), (**C**) extrinsic epigenetic age acceleration (*EEAA*). The multivariate Cox regression model included the following additional covariates: chronological age, body mass index (category), educational level (category), alcohol intake, smoking pack years, prior history of diabetes, prior history of cancer, hypertension status, recreational physical activity (category). The rows correspond to the different cohorts. Each row depicts the hazard ratio and a 95% confidence interval. The coefficient estimates from the respective studies were meta-analyzed using a fixed-effect model weighted by inverse variance (implemented in the *metafor* R package [34]). The sub-title of each plot reports the meta-analysis p-value and a heterogeneity test p-value (Cochran's Q-test).

All considered measures of epigenetic age acceleration were predictive of time to death in univariate Cox models (p_*AgeAccel*_=1.9×10^−11^, p_*IEAA*_=8.2×10^−9^, p_*EEAA*_=7.5×10^−43^, Figure 2) and multivariate Cox models adjusting for risk factors and pre-existing disease status (p_*AgeAccel*_=5.4×10^−5^, p_*IEAA*_=5.0×10^−4^, p_*EEAA*_=3.4×10^−19^, Figure 3).

### Interpreting effect sizes and variance of epigenetic age acceleration

Subjects differed substantially in terms of their measures of epigenetic age acceleration, e.g. *EEAA* ranged from −28 to 28 years in the WHI (standard deviation =6.4 years, Supplementary Table 3).

About five percent of the participants of the WHI exhibited an *EEAA* value larger than 10, which is associated with a 48% increased hazard of death as can be seen from the following calculation. The HR of *EEAA* is 1.040 if EEAA=1 (Figure 2c) but it is HR=1.48=(1.040)^10^ if EEAA=10. Negative values of age acceleration were associated with a lower hazard of mortality. For example, 20% of subjects had an *EEAA* value less than −5, which is associated with an 18% decrease in the hazard of death (HR=0.82=1.04^−5^).

### Subgroup analysis

With few exceptions, we found that the associations between *EEAA* and time to death remained highly significant in subgroups stratified by race, sex, follow-up duration, body mass index, smoking status, physical activity (Table 3) and in subgroups stratified by prevalent disease at baseline such as cancer, coronary artery disease, hypertension and type 2 diabetes (Table 4). Only one subgroup led to an insignificant finding (p>0.05) in our univariate model analysis: namely subjects with less than 5 years of follow up (Table 3). For multivariate models, we failed to observe significant associations for the following subgroups: i) less than 5 years of follow up, ii) between 5 and 10 years of follow up, iii) current smokers, iv) obese individuals, v) Hispanics, vi) individuals with cancer, and vii) subjects with coronary artery disease. The insignificant results in multivariate models in cancer patients or CAD patients might reflect the relatively low sample sizes or that epigenetic age acceleration is dwarfed by other predictors of mortality in subjects with severe diseases. Hazard ratio estimates remained highly consistent across all subgroups examined.

**Table 3:** Subgroup analysis by demographic factors

	Age-adjusted		Full model	
Subgroup	HR	*p*-value	HR	*p*-value
***Race***				
White	1.05	3.0×10^−26^	1.03	1.3×10^−5^
Black	1.04	7.8×10^−20^	1.02	7.6×10^−3^
Hispanic	1.05	1.1×10^−2^	1.06	5.3×10^−2^
***p_interaction_***		0.62		0.14
***Sex***				
Men	1.04	7.1×10^−15^	1.03	1.9×10^−2^
Women	1.04	3.7×10^−10^	1.03	1.9×10^−5^
***p_interaction_***		0.63		0.95
***Follow-up duration***				
<5 years	1.02	0.20	0.98	0.79
5-10 years	1.02	1.8×10^−3^	1.02	0.17
>10 years	1.03	4.5×10^−9^	1.02	4.1×10^−2^
***p_interaction_***		0.67		0.84
***BMI categories***				
Underweight	1.11	9.4×10^−3^	1.04	8.9×10^−3^
Normal	1.06	6.1×10^−19^	1.04	2.3×10^−2^
Overweight	1.04	1.46×10^−8^	1.03	5.0×10^−2^
Obese	1.04	2.2×10^−11^	1.02	7.1×10^−2^
***p_interaction_***		0.05		0.75
***Smoking status***				
Never	1.03	6.9×10^−6^	1.04	4.8×10^−3^
Former	1.05	4.2×10^−22^	1.03	6.3×10^−4^
Current	1.06	2.1×10^−4^	1.01	0.47
***p_interaction_***		0.05		0.20
***Physical activity status***				
Yes	1.05	3.8×10^−6^	1.02	1.9×10^−3^
No	1.03	2.5×10^−2^	1.03	2.2×10^−2^
***p_interaction_***		0.23		0.65

Age-adjusted and fully adjusted associations for *EEAA* to all-cause mortality by subgroup (rows). The fully adjusted model includes the following covariates: body mass index, educational level, alcohol intake, smoking pack-years, prior history of diabetes, prior history of cancer, hypertension status, self-reported recreational physical activity.

**Table 4:** StSubgroup analysis by prevalent disease status

	Age-adjusted		Full model	
Subgroup	HR	*p*-value	HR	*p*-value
***Cancer status***				
Yes	1.05	2.5×10^−10^	1.02	0.18
No	1.05	2.3×10^−13^	1.03	1.7×10^−4^
***p_interaction_***		0.92		0.73
***Coronary artery disease status***				
Yes	1.04	2.4×10^−5^	1.01	0.60
No	1.04	1.5×10^−12^	1.02	1.5×10^−4^
***p_interaction_***		0.43		0.99
***Hypertension status***				
Yes	1.04	7.4×10^−17^	1.03	2.9×10^−3^
No	1.05	7.1×10^−6^	1.02	8.6×10^−3^
***p_interaction_***		0.41		0.45
***Type 2 diabetes status***				
Yes	1.04	8.6×10^−13^	1.03	1.7×10^−3^
No	1.04	1.2×10^−10^	1.02	9.3×10^−3^
***p_interaction_***		0.70		0.25

Age-adjusted and fully adjusted associations for *EEAA* to all-cause mortality in different subgroups (rows). The fully adjusted model includes the following covariates: body mass index, educational level, alcohol intake, smoking pack-years, prior history of diabetes, prior history of cancer, hypertension status, self-reported recreational physical activity.

We did not observe significant differences in the estimated hazard ratios across any subgroup (Tables 3 and 4). Specifically, racial/ethnic differences in HR were not observed (interaction p=0.62 in age-adjustment models and p=0.14 in full models). Overall, these subgroup analysis results confirm that epigenetic age acceleration is an independent predictor of earlier mortality even after adjusting for possible confounders and within major subgroups of the population.

### Hazard ratio of death versus follow up time and median age

**Figure 4:**
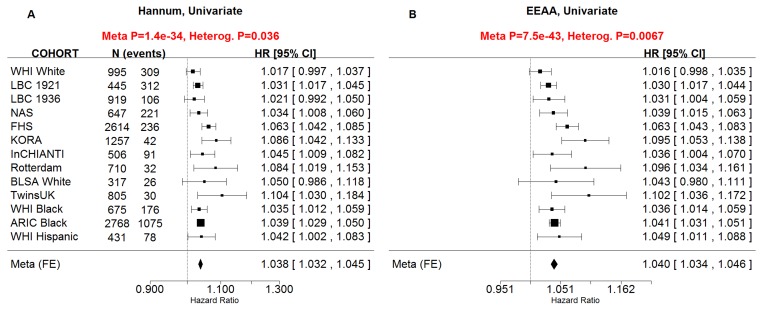
Hazard ratio of death versus cohort characteristics Each circle corresponds to a cohort (data set). Circle sizes correspond to the square root of the number of observed deaths, because the statistical power of a Cox model is determined by the number of observed deaths. (**A**-**C**) The y-axis of each panel corresponds to the natural log of the hazard ratio (ln HR) of a univariate Cox regression model for all-cause mortality. Each panel corresponds to a different measure of epigenetic age acceleration (**A**) universal age acceleration, (**B**) intrinsic age acceleration, (**C**) extrinsic age acceleration. Panels (**D**-**F**) are analogous to those in A-C but the x-axis corresponds to the median age of the subjects at baseline (Table 1). The title of each panel reports the Wald test statistic (*T*) and corresponding p-value resulting from a weighted linear regression model (y regressed on x) where each point (data set) is weighted by the square root of the number of observed deaths. The dotted red line represents the regression line. The black solid line represents the line of identify (i.e., no association).

The large number of cohorts allowed us to relate cohort characteristics (such as median age or median follow up removing time) to strength of association with mortality. We did not find a statistically significant relationship between the hazard ratio of death for the median age of the cohort or the follow up time (Figure 4).

### Robustness analysis

To assess the robustness of our findings, we also carried out a leave-one-out analysis by re-running the metaanalysis after removing data from individual cohorts. The resulting p-values are highly robust with respect to a single data set from the analysis (Supplementary Table 5). In our study, we used a fixed effects meta-analysis method for the sake of consistency with previous analyses [5]. However, our results remain qualitatively the same after using a random effects meta-analysis method (Supplementary Figure 4).

## DISCUSSION

The current study corroborates previous findings regarding the predictive power of DNA methylation-based biomarkers of age for mortality [5,6,8]. We further examined novel variants of these measures that are either independent of blood cell counts or are enhanced by changes in blood cell sub-populations. We showed that the extrinsic measure *EEAA* out-performs previous measures of age acceleration when it comes to predicting all-cause mortality. Furthermore, the associations between epigenetic age acceleration and mortality did not differ significantly across subgroups of race/ethnicity, sex, BMI, smoking status, physical activity status, or major chronic diseases. The consistency of the associations across multiple subgroups lends support to the notion that epigenetic age acceleration captures some aspect of biological aging over and above chronological age and other risk factors.

The development of suitable measures of biological age has been a key goal in the field of aging research [13]. Many biomarkers of age have been posited including epigenetic alterations of the DNA (e.g., DNA methylation), transcriptomic changes in blood [14], telomere length [15], whole-body function such as gait speed (reviewed in [16]). The current study does not aim to replace existing blood based biomarkers, but rather, we aimed to demonstrate that it complements existing markers. Above all, this study shows that epigenetic age captures an aspect of biological age, as assessed through lifespan, above and beyond chronological age, blood cell composition, and a host of traditional risk factors of mortality.

The measures of epigenetic age acceleration are attractive because they are highly robust and because their measurement only involve DNA methylation data. While actual flow cytometry data will always be preferable to imputed blood cell count data (based on DNA methylation data), the measures of age acceleration do not require the measurement of flow data. Rather, measures of intrinsic and extrinsic epigenetic age used blood cell count estimates resulting from DNA methylation data. The measure of extrinsic age acceleration EEAA reflects aspects of immuno-senescence because, by construction, it correlates with age-related changes in blood cell composition, such as T lymphocyte populations, which underlie much of the age-related decline in the protective immune response [9–12]. Thus, the high predictive significance of *EEAA* for all-cause mortality probably reflects the fact that it assesses multiple aspects of the biological age of the immune system including both changes in blood cell composition and cell-intrinsic epigenetic changes. It has been known for decades that poor T cell functioning is predictive of mortality [17].

The findings surrounding the predictive utility of intrinsic epigenetic age acceleration are biologically compelling and point to a new frontier in aging research. Our study strongly suggests *IEAA* is reflective of an intrinsic epigenetic clock that is associated with mortality independent of chronological age, changes in blood cell composition, and traditional risk factors of mortality. *IEAA* probably captures a cell-type independent component of the aging process for the following reasons. First, *IEAA* is moderately preserved across different tissues and cell types collected from the same subject (Figure 1). Second, *IEAA* but not *EEAA* is predictive of lung cancer [18]. Third, only *IEAA* and *AgeAccel* relate to centenarian status [8].

Overall, our results inform the ongoing debate about whether epigenetic biomarkers of age capture an aspect of biological age. While epigenetic processes are unlikely to be the only mediators of chronological age on mortality—in fact, multiple risk factors have stronger effects on mortality—our results suggest that at least one of the mediating processes relates to the epigenetic age of blood tissue and that this process is independent of age-dependent changes in blood cell composition. Future studies will be useful for gaining a mechanistic understanding of this intrinsic epigenetic aging process.

## MATERIALS AND METHODS

### Measures of epigenetic age

We used an epigenetic biomarker of age based on 353 CpG markers as one measure of epigenetic age because: a) it is an accurate measurement of age across multiple tissues [3]; b) we previously showed that it is predictive of all-cause mortality [5]; c) it correlated with measures of cognitive/physical fitness and neuro-pathology in the elderly [19,20]; and d) it was associated with conditions that are of interest in aging research including Down's syndrome [21], Huntington's disease [22], Parkinson's disease [23], obesity [24], HIV infection [25], menopause [26], centenarian status [27], ethnicity and sex [28], and cellular senescence [3,29]. This epigenetic age estimator not only lends itself to measuring aging effects in elderly subjects; but also applies to prenatal brain samples [30] and blood samples from minors [31]. Epigenetic age is defined as the predicted value of age based on the DNA methylation levels of 353 CpGs. Mathematical details and software tutorials for estimating epigenetic age can be found in the additional files of [3]. All of the described epigenetic measures of aging and age acceleration are implemented in our freely available software (https://dnamage.genetics.ucla.edu) [3].

### DNA methylation age estimate by Hannum et al (2013)

We also used an alternative measure of epigenetic age developed by Hannum et al (2013) [2]. The resulting age estimate is based on the 71 CpGs and coefficient values from the third supplementary table [2]. The authors developed this age prediction method by using an elastic net regression model for predicting chronological age based on DNA methylation levels from whole blood.

### Measures of epigenetic age acceleration

Table 2 provides an overview of our measures of epigenetic age acceleration. The universal measure of age acceleration (*AgeAccel*), which is valid for a wide range of tissue types, is defined as the residual resulting from a linear regression model that regresses the Horvath estimate of epigenetic age on chronological age. Thus, a positive value for *AgeAccel* indicates that the observed epigenetic age is higher than that predicted, based on chronological age. *AgeAccel* has a relatively weak correlation with blood cell counts [25], but it still relates to estimated blood cell counts, as seen in Supplementary Table 4.

To estimate “pure” epigenetic aging effects that are not influenced by differences in blood cell counts (“intrinsic” epigenetic age acceleration, *IEAA*), we obtained the residual resulting from a multivariate regression model of epigenetic age on chronological age and various blood immune cell counts (naive CD8+ T cells, exhausted CD8+ T cells, plasmablasts, CD4+ T cells, natural killer cells, monocytes, and granulocytes) imputed from methylation data.

Extrinsic epigenetic age acceleration measures capture both cell intrinsic methylation changes and extracellular changes in blood cell composition. Our measure of *EEAA* is defined using the following three steps. First, we calculated the epigenetic age measure from Hannum et al [2], which already correlated with certain blood cell types [5]. Second, we increased the contribution of immune blood cell types to the age estimate by forming a weighted average of Hannum's estimate with 3 cell types that are known to change with age: naïve (CD45RA+CCR7+) cytotoxic T cells, exhausted (CD28-CD45RA-) cytotoxic T cells, and plasmablasts using the Klemera-Doubal approach [32]. The weights used in the weighted average are determined by the correlation between the respective variable and chronological age [32]. The weights were chosen on the basis of the WHI data. Thus, the same (static) weights were used for all data sets. *EEAA* was defined as the residual variation resulting from a univariate model regressing the resulting age estimate on chronological age. By construction, *EEAA* is positively correlated with the estimated abundance of exhausted CD8+ T cells, plasmablast cells, and a negative correlated with naive CD8+ T cells. Blood cell counts were estimated based on DNA methylation data as described in the next section. By construction, the measures of *EEAA* track both age related changes in blood cell composition and intrinsic epigenetic changes. None of our four measures of epigenetic age acceleration are correlated with chronological age.

### Estimating blood cell counts based on DNA methylation levels

We estimate blood cell proportions using two different software tools. Houseman's estimation method [33], which is based on DNA methylation signatures from purified leukocyte samples, was used to estimate the proportions of cytotoxic (CD8+) T cells, helper (CD4+) T, natural killer, B cells, and granulocytes. The software does not allow us to identify the type of granulocytes in blood (neutrophil, eosinophil, or basophil) but we note that neutrophils tend to be the most abundant granulocyte (∼60% of all blood cells compared with 0.5-2.5% for eosinophils and basophils). To estimate the percentage of exhausted CD8+ T cells (defined as CD28-CD45RA-), plasmablasts, and the number (count) of naïve CD8+ T cells (defined as CD45RA+CCR7+), we used the “Horvath method” [25], which is implemented in the advanced analysis option of the epigenetic age calculator software [3]. We and others have shown that imputed blood cell counts have moderately high correlations with corresponding flow cytometric data, e.g. r=0.86 for naïve CD4+ T cells, r=0.68 for naïve CD8+T, and r=0.49 for exhausted CD8+ T cells [28].

### Cox regression models and meta-analysis

Here, we used Cox models for analyzing the censored survival time data (from the age at blood draw until age at death or last follow-up). We regressed the censored survival times on covariates using Cox regression models implemented in the R function *coxph* in the *survival* package. The resulting coefficient values (interpreted as log hazard ratios) and standard errors were combined using the R software package *metafor* [34]. The meta-analysis was carried out with the R command *rma* (with arguments *method=“FE”* to get fixed effects estimates). The forest plots were created using the R function *forest* (with argument *atransf=exp* to exponentiate the estimate of the log hazard ratios).

### Sample exclusions

In addition to cohort-specific quality checks, we further excluded individuals who had ever been diagnosed with leukemia (ICD-9: 203-208), reported receiving chemotherapy, and whose methylation beta value distributions deviated substantially from a gold standard (according to the quality statistic *corSampleVSgold standard*<0.80 from the online age calculator [35–37]).

## SUPPLEMENTARY DATA TABLES AND FIGURES


