# Supplementary material for: DNA methylation-based measures of biological age: meta-analysis predicting time to death
Source: Aging (Albany NY). 2016 Sep 28;8(9):1844–59. doi: 10.18632/aging.101020 (PMC5076441; doi:10.18632/aging.101020)
Supplement: Supplementary file 1 [file aging-08-1844-s001.pdf]

SUPPLEMENTARY DATA

Supplementary Table 1. Average pairwise correlations between chronological age, *epigenetic age* based on the Horvath method, and *epigenetic age* based on Hannum.

	Age	*Epigenetic age* (Horvath)	*Epigenetic age* (Hannum)
**Age**	-	0.74	0.81
***Epigenetic age* (Horvath)**	0.74	-	0.76
***Epigenetic age* (Hannum)**	0.81	0.76	-

Supplementary Table 2. Correlations between estimated blood cell abundances and chronological age.

Cohort	Plasmablasts	CD8+CD28-CD45RA-	CD8+ naive	CD4+ naive	CD8+ total	CD4+ total	NK cells	B cells	Monocytes	Granulocytes
ARIC	0.03	0.16	-0.22	-0.13	-0.06	-0.08	0.13	-0.08	0.09	0.04
FHS	0.23	0.36	-0.38	-0.22	-0.19	-0.23	0.20	-0.27	0.17	0.16
InCHIANTI	0.06	0.32	-0.43	-0.34	-0.19	-0.20	0.23	-0.20	0.08	0.11
KORA	0.20	0.48	-0.53	-0.37	-0.07	-0.37	0.12	-0.22	0.20	0.16
LBC1921	0.03	0.03	-0.14	-0.09	0.10	0.01	0.05	-0.07	0.10	-0.07
LBC1936	0.11	0.04	-0.05	-0.09	-0.03	0.00	-0.05	-0.13	-0.09	0.05
NAS	0.04	0.28	-0.21	-0.07	0.01	-0.16	0.12	-0.10	-0.01	0.08
RSIII	0.11	0.20	-0.26	-0.16	-0.10	-0.14	0.06	-0.16	0.14	0.11
TwinsUK	-0.02	0.21	-0.35	-0.08	-0.14	0.07	0.27	-0.02	0.10	-0.14
WHI White	0.09	0.21	-0.17	-0.13	-0.13	-0.10	0.17	-0.12	0.10	0.04
WHI Black	0.12	0.24	-0.21	-0.13	-0.07	-0.17	0.16	-0.18	0.11	0.07
WHI Hispanic	0.08	0.26	-0.21	-0.15	-0.11	-0.13	0.17	-0.10	0.03	0.08
**MEAN**	**0.09**	**0.23**	**-0.26**	**-0.16**	**-0.08**	**-0.12**	**0.14**	**-0.14**	**0.09**	**0.06**

The values shown are robust correlation coefficients (biweight midcorrelation, which is based on medians). Colors reflect the direction and magnitude of the correlation coefficients (blue=negative correlation, red=positive correlation).

Supplementary Table 3. Descriptive statistics of measures of epigenetic age acceleration by cohort.

Epigenetic age acceleration measure	Cohort	SD	Min	25th percentile	Median (50th percentile)	75th percentile	Max
AgeAccel	ARIC	5.081	-34.380	-3.191	-0.100	3.220	25.620
AgeAccel	FHS	4.621	-16.490	-3.110	-0.367	2.460	35.160
AgeAccel	InCHIANTI	4.999	-33.600	-3.084	-0.338	2.223	29.770
AgeAccel	KORA	4.937	-24.810	-3.423	-0.117	2.905	20.660
AgeAccel	LBC1921	6.971	-24.240	-3.884	-0.170	3.943	39.730
AgeAccel	LBC1936	6.485	-30.160	-3.879	0.116	3.801	42.100
AgeAccel	NAS	5.365	-16.930	-3.706	-0.498	2.909	32.830
AgeAccel	RSIII	6.003	-18.570	-4.325	-0.046	4.276	20.050
AgeAccel	TwinsUK	4.108	-13.760	-2.514	0.274	2.899	13.560
AgeAccel	WHI (white)	5.153	-22.560	-2.843	-0.103	3.443	22.790
AgeAccel	WHI (Black)	6.091	-21.900	-5.424	-1.977	1.824	39.930
AgeAccel	WHI (Hispanic)	4.494	-14.080	-3.831	-0.535	2.458	14.790
AgeAccel	BLSA	4.828	-11.620	-2.966	0.290	3.197	25.180
AgeAccel	ARIC	5.914	-38.770	-3.766	0.105	3.648	39.350
AgeAccelHannum	FHS	5.279	-23.480	-3.303	-0.174	3.001	36.680
AgeAccelHannum	InCHIANTI	6.028	-43.640	-3.152	0.431	3.573	31.490
AgeAccelHannum	KORA	4.996	-30.280	-3.266	-0.314	2.711	37.410
AgeAccelHannum	LBC1921	7.203	-25.140	-4.574	-0.821	3.722	51.840
AgeAccelHannum	LBC1936	6.670	-27.520	-4.172	0.131	4.183	31.650
AgeAccelHannum	NAS	5.161	-12.250	-3.310	-0.787	2.511	22.750
AgeAccelHannum	RSIII	6.090	-18.600	-3.825	0.115	4.152	17.970
AgeAccelHannum	TwinsUK	5.246	-17.040	-2.949	0.320	3.821	20.260
AgeAccelHannum	WHI (white)	5.557	-23.460	-3.644	-0.086	3.441	21.760
AgeAccelHannum	WHI (Black)	6.317	-23.490	-4.773	-0.891	3.045	31.480
AgeAccelHannum	WHI (Hispanic)	5.357	-12.740	-2.139	1.133	4.469	20.880
AgeAccelHannum	BLSA	5.709	-15.720	-2.942	0.429	3.801	31.730
AgeAccelHannum	ARIC	4.928	-34.020	-3.010	-0.057	3.068	23.810
IEAA	FHS	4.491	-16.010	-2.901	-0.199	2.547	32.160
IEAA	InCHIANTI	4.783	-30.930	-2.898	-0.374	2.364	29.470
IEAA	KORA	4.647	-29.350	-3.276	-0.114	2.761	18.740
IEAA	LBC1921	6.228	-22.890	-3.664	0.135	3.554	24.600
IEAA	LBC1936	6.162	-26.310	-3.596	0.035	3.718	34.090
IEAA	NAS	4.929	-24.190	-3.019	-0.458	2.694	22.500
IEAA	RSIII	5.130	-16.610	-3.330	-0.026	3.387	15.190
IEAA	TwinsUK	4.016	-14.590	-2.257	0.234	2.718	13.100
IEAA	WHI (white)	4.797	-21.460	-2.608	0.183	3.287	21.440

IEAA	WHI (Black)	5.588	-20.320	-3.202	0.067	3.197	42.660
IEAA	WHI (Hispanic)	4.333	-13.520	-3.971	-1.397	1.864	12.480
IEAA	BLSA	4.488	-10.370	-2.658	0.017	3.330	23.120
EEAA	ARIC	6.673	-32.940	-4.096	0.135	4.149	38.560
EEAA	FHS	5.800	-26.100	-3.607	0.113	3.315	38.250
EEAA	InCHIANTI	6.710	-44.360	-3.327	0.576	4.236	33.630
EEAA	KORA	5.405	-26.610	-3.526	-0.339	3.189	37.600
EEAA	LBC1921	7.745	-21.180	-5.262	-0.973	4.306	52.120
EEAA	LBC1936	7.116	-30.590	-4.439	0.168	4.589	31.530
EEAA	NAS	5.596	-13.730	-3.443	-0.579	2.987	23.540
EEAA	RSIII	6.861	-22.380	-4.392	0.267	4.794	21.080
EEAA	TwinsUK	5.840	-22.790	-3.409	0.401	4.023	23.020
EEAA	WHI (white)	6.089	-22.710	-3.888	0.015	4.008	22.320
EEAA	WHI (Black)	6.906	-27.600	-5.735	-1.382	2.827	27.900
EEAA	WHI (Hispanic)	5.779	-14.310	-1.679	2.371	5.651	23.450
EEAA	BLSA	6.256	-18.040	-3.435	0.921	4.724	29.230

Supplementary Table 4. Pairwise correlations (mean and standard error (SE) across cohorts) between blood cell counts estimated from DNA methylation profiles (rows) and several measures of epigenetic age acceleration (columns).

**BloodCell**	***AgeAccel*** **average *r* (SE)**	***IEAA*** **average *r* (SE)**	***EEAA*** **average *r* (SE)**	***AgeAccel$_{Hannum}$*** **average *r* (SE)**	***IEAA.Hannum*** **average *r* (SE)**
Plasma Blast	0.02 (0.031)	0 (0.002)	0.28 (0.034)	0.20 (0.033)	0 (0.018)
Exhausted CD8+	0.18 (0.039)	0 (0.009)	0.50 (0.033)	0.29 (0.044)	0 (0.046)
CD8.naive	-0.18 (0.043)	0 (0.011)	-0.52 (0.04)	-0.35 (0.048)	0 (0.05)
CD4.naive	-0.09 (0.033)	0.07 (0.017)	-0.36 (0.038)	-0.28 (0.041)	0.06 (0.046)
CD8T	0.19 (0.026)	0 (0.023)	0 (0.046)	0 (0.041)	-0.01 (0.024)
CD4T	-0.20 (0.032)	0 (0.004)	-0.46 (0.034)	-0.34 (0.036)	0 (0.026)
NK	0.13 (0.026)	0 (0.002)	0.17 (0.042)	0.10 (0.042)	0 (0.03)
Bcell	-0.08 (0.051)	-0.11 (0.028)	-0.05 (0.068)	-0.01 (0.061)	-0.02 (0.035)
Monocyte	0.05 (0.026)	0 (0.006)	0.12 (0.042)	0.07 (0.04)	0 (0.019)
Granulocyte	-0.03 (0.033)	0 (0.005)	0.16 (0.049)	0.14 (0.042)	0 (0.018)

AgeAccel=univeral measure of age acceleration based on Horvath estimate. *IEAA*=intrinsic epigenetic age acceleration based on the Horvath estimate. *EEAA* = extrinsic epigenetic age acceleration which is an enhanced version of the Hannum estimate. *AgeAccel$_{Hannum}$*=univeral measure of age acceleration based on the Hannum estimate. *IEAA.Hannum*=intrinsic epigenetic age acceleration based on Hannum estimate. By design, the intrinsic measures have only weak correlations with blood cell counts. By contrast, *AgeAccel$_{Hannum}$* and *EEAA* have moderately strong correlations with blood cell counts.

Supplementary Table 5. Leave-one-out analysis by cohort for relating *EEAA* to time to death.

**Cohort removed**	**Age-adjusted model**	**Fully adjusted model**
(None)	1.04 ($p=1.81x10^{-42}$)	1.02 ($p=1.94x10^{-7}$)
ARIC	1.04 ($p=1.00x10^{-26}$)	1.03 ($p=1.09x10^{-6}$)
FHS	1.04 ($p=1.23x10^{-34}$)	1.02 ($p=5.09x10^{-6}$)
LBC 1921	1.04 ($p=1.17x10^{-38}$)	1.02 ($p=3.21x10^{-6}$)
LBC 1936	1.04 ($p=1.94x10^{-41}$)	1.02 ($p=1.39x10^{-7}$)
WHI (Whites)	1.04 ($p=2.61x10^{-43}$)	1.02 ($p=2.77x10^{-7}$)
WHI (Blacks)	1.04 ($p=3.46x10^{-40}$)	1.02 ($p=7.42x10^{-7}$)
WHI (Hispanics)	1.04 ($p=4.29x10^{-41}$)	1.02 ($p=6.17x10^{-7}$)
NAS	1.04 ($p=3.91x10^{-40}$)	1.02 ($p=2.42x10^{-7}$)
InCHIANTI	1.04 ($p=1.98x10^{-41}$)	1.02 ($p=7.38x10^{-7}$)
Rotterdam	1.04 ($p=4.35x10^{-41}$)	1.02 ($p=2.16x10^{-7}$)
KORA	1.04 ($p=1.93x10^{-39}$)	1.02 ($p=3.76x10^{-7}$)
TwinsUK	1.04 ($p=3.76x10^{-41}$)	1.02 ($p=4.66x10^{-7}$)

The table reports hazards ratios and corresponding p-values based on a Cox regression. The fully adjusted model includes the following covariates: body mass index, educational level, alcohol intake, smoking pack-years, prior history of diabetes, prior history of cancer, hypertension status, self-reported recreational physical activity.

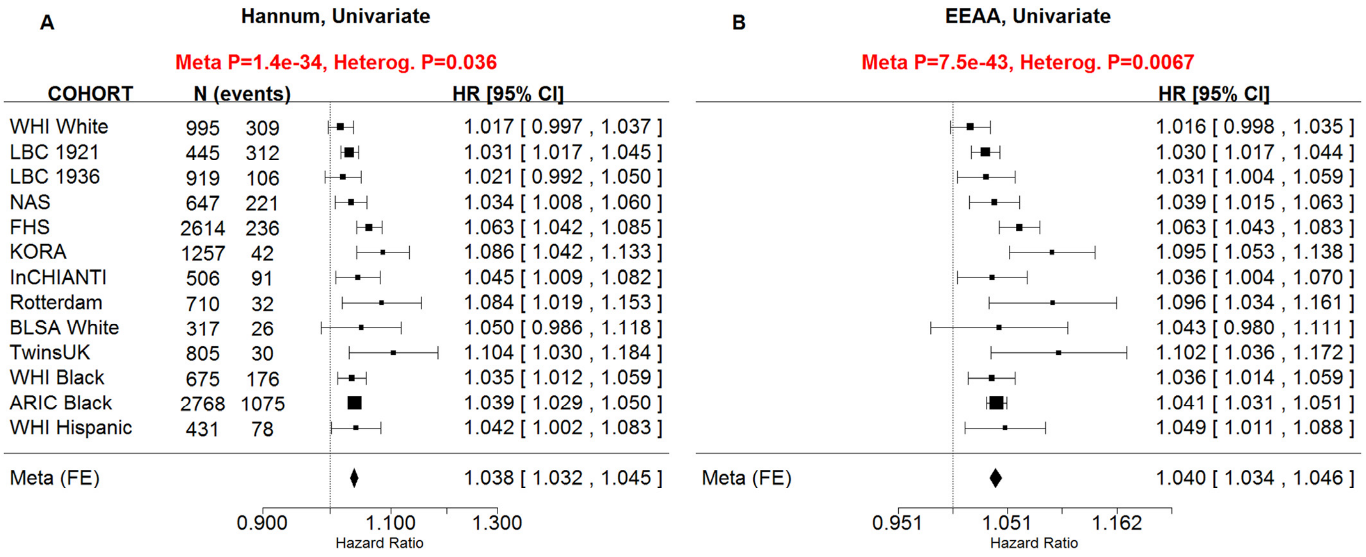

Supplementary Figure 1. Univariate Cox regression model analysis of all-cause mortality, contrasting existing and novel measures of age acceleration. The rows correspond to the different cohorts. Each row depicts the hazard ratio and a 95% confidence interval. To combine the coefficient estimates from the respective studies into a single estimate, we applied a fixed-effect model weighted by inverse variance (implemented in the metafor R package [30]). (**A**) This measure of age acceleration is based on Hannum et al [1]. Specifically, we estimated the age using the 71 CpGs and coefficient values from Hannum. Next, the measure of age acceleration was defined as residuals resulting from regressing the epigenetic age estimate on chronological age. (**B**) Extrinsic epigenetic age acceleration (EEAA). The sub-title of each plot reports the meta-analysis p-value and a heterogeneity test p-value (Cochran's Q-test). It is not appropriate to compare the hazard ratios and confidence intervals of the different measures directly because the measures have different scales/distributions. However, it is appropriate to compare the meta-analysis p-values (colored in red).

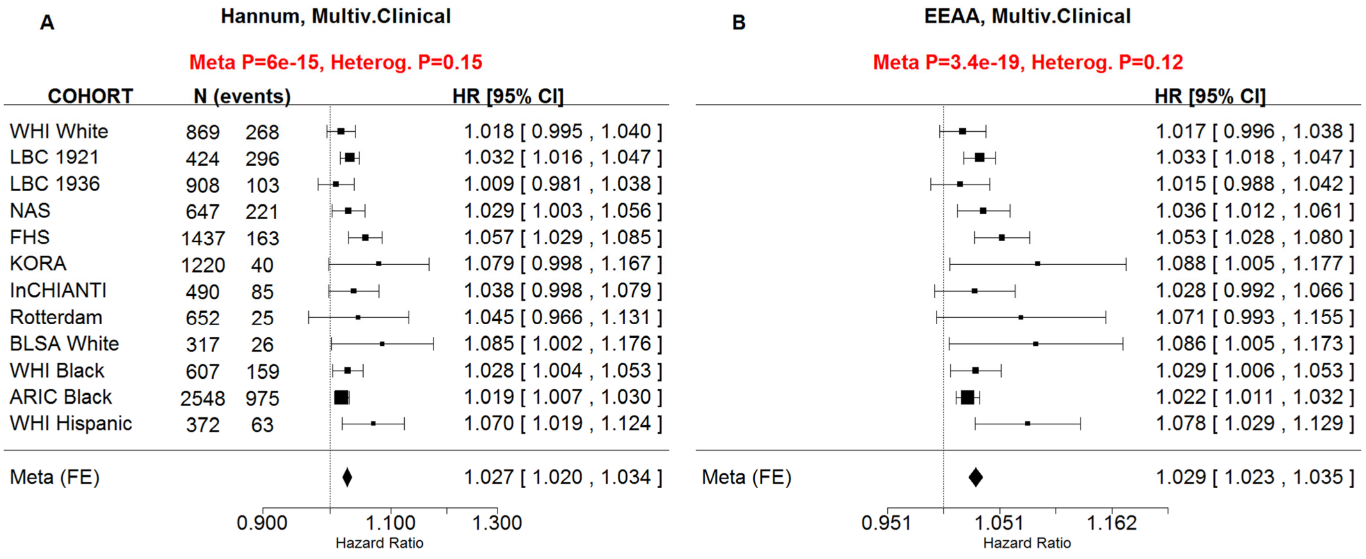

Supplementary Figure 2. Multivariate Cox regression model analysis of all-cause mortality, contrasting existing and novel measures of age acceleration. The multivariate Cox regression model included the following additional covariates: chronological age, body mass index (category), educational level (category), alcohol intake, smoking pack years, prior history of diabetes, prior history of cancer, hypertension status, recreational physical activity (category). The rows correspond to separate cohorts. Each row depicts the hazard ratio (HR) and a 95% confidence interval. (**A**) Age acceleration based on Hannum et al [6], (**B**) Extrinsic epigenetic age acceleration (EEAA). The sub-title of each plot reports the meta-analysis p-value and a heterogeneity test p-value (Cochran's Q-test).

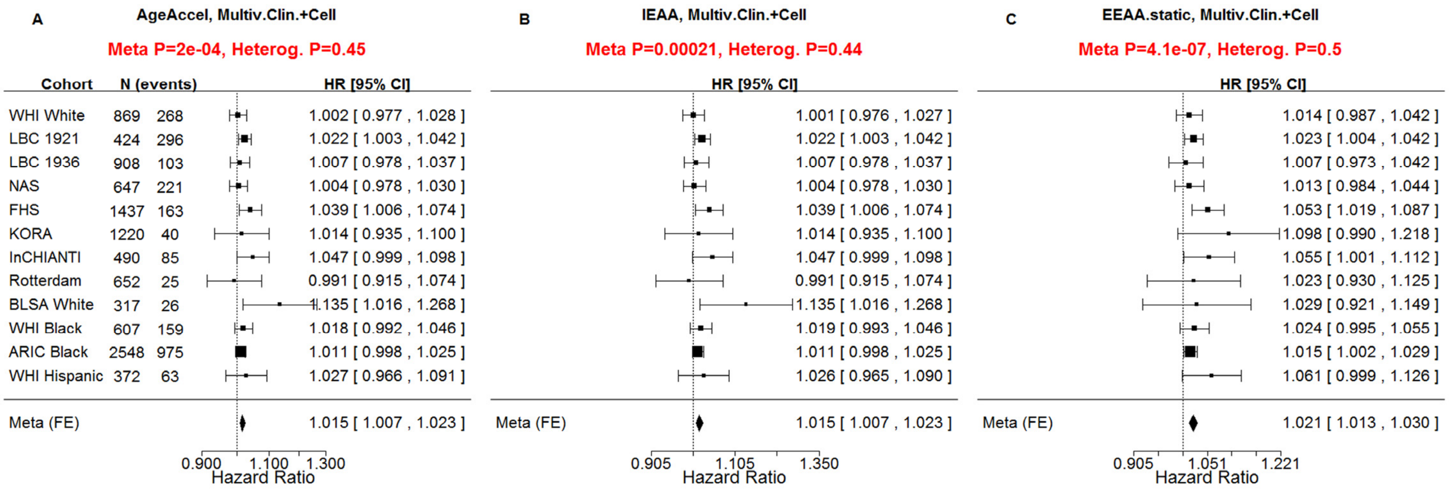

Supplementary Figure 3. Multivariate Cox regression analysis of all-cause mortality adjusted for blood cell counts and clinical covariates. A multivariate Cox regression model was used to relate the censored survival time (time to all-cause mortality) to (**A**) the universal measure of age acceleration (AgeAccel), (**B**) intrinsic epigenetic age acceleration (IEAA), (**C**) extrinsic epigenetic age acceleration (EEAA). The multivariate Cox regression model included blood cell counts (exhausted CD8+ T cells, naïve CD8+, CD4+ T cells, natural killer, monocytes, granulocytes, and plasmablasts) and clinical covariates (chronological age, body mass index, educational level, alcohol intake, smoking pack years, prior history of diabetes, prior history of cancer, hypertension status, recreational physical activity). The rows correspond to the different cohorts. Each row depicts the hazard ratio (HR) and a 95% confidence interval. Estimates were meta-analyzed using a fixed-effect model weighted by inverse variance. The sub-title of each plot reports the meta-analysis p-value and a heterogeneity test p-value (Cochran's Q-test).

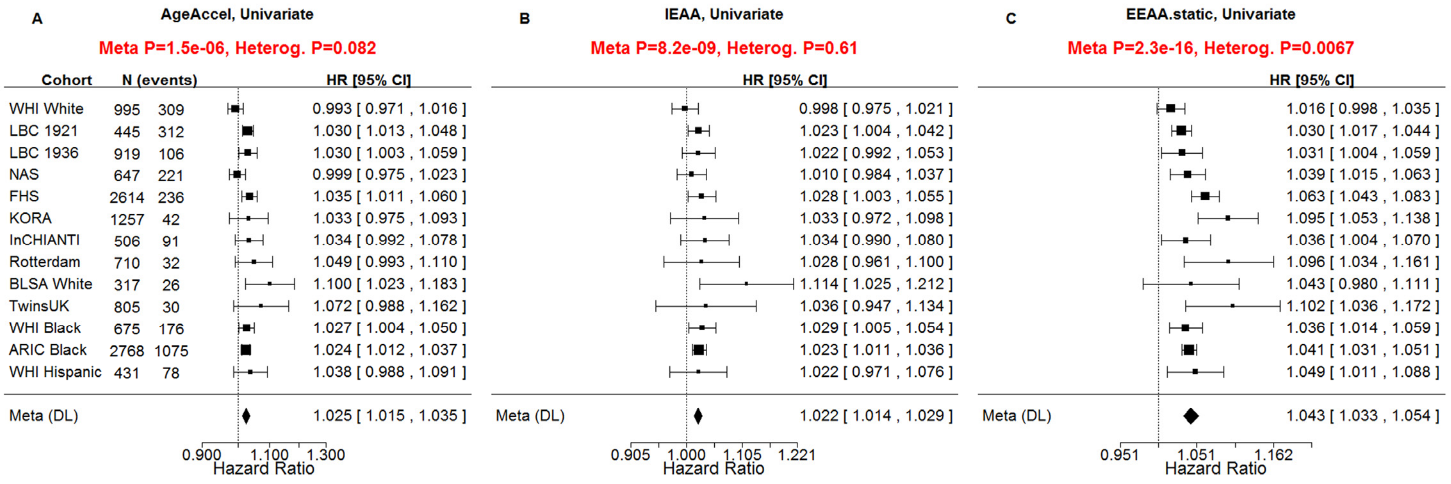

Supplementary Figure 4. Random effects meta-analysis for univariate Cox models. The figure is analogous to Figure 2 in our article except that it uses a random-effects meta-analysis (DerSimonian-Laird) instead of a fixed-effects model. A univariate Cox regression model was used to relate the censored survival time (time to all-cause mortality) to (**A**) the universal measure of age acceleration (AgeAccel), (**B**) intrinsic epigenetic age acceleration (IEAA), (**C**) extrinsic epigenetic age acceleration (EEAA). To combine the coefficient estimates from the respective studies into a single estimate, we applied the DerSimonian-Laird random effects model. The sub-title of each plot reports the meta-analysis p-value and a heterogeneity test p-value (Cochran's Q-test).
